# A methodology to establish a database to study gene environment interactions for childhood asthma

**DOI:** 10.1186/1471-2288-10-107

**Published:** 2010-12-06

**Authors:** Stephen W Turner, Jon G Ayres, Tatiana V Macfarlane, Anil Mehta, Gita Mehta, Colin N Palmer, Steve Cunningham, Tim Adams, Krishnan Aniruddhan, Claire Bell, Donna Corrigan, Jason Cunningham, Andrew Duncan, Gerard Hunt, Richard Leece, Una MacFadyen, Jonathan McCormick, Sally McLeish, Andrew Mitra, Deborah Miller, Elizabeth Waxman, Alan Webb, Slawomir Wojcik, Somnath Mukhopadhyay, Donald Macgregor

**Affiliations:** 1 Academic Child Health, University of Aberdeen, Aberdeen, UK; 2 Institute of Occupational and Environmental Medicine, University of Birmingham, Birmingham, UK; 3 Division of Applied Medicine, University of Aberdeen, Aberdeen, UK; 4 Maternal and Child Health Services, University of Dundee, Dundee, UK; 5 Population Pharmacogenetics Group, Biomedical Research Institute, University of Dundee, Dundee, UK; 6 Department of Respiratory, Sleep and General Medicine, Royal Hospital for Sick Children Edinburgh, Edinburgh, UK; 7 Department of Paediatrics, Crosshouse Hospital, Kilmarnock, UK; 8 Department of Paediatrics, Victoria Hospital, Kirkcaldy, UK; 9 Department of Paediatrics, Wishaw General Hospital, Wishaw, UK; 10 Academic Department of Paediatrics, Brighton and Sussex Medical School, Brighton, UK; 11 Department of Paediatrics, Borders General Hospital, Melrose, UK; 12 Department of Paediatrics, Royal Alexandra Hospital, Paisley, UK; 13 Department of Paediatrics, Stirling Royal Infirmary, Stirling, UK; 14 Women & Child Health, Tayside Children's Hospital, Ninewells Hospital, Dundee, UK; 15 Department of Paediatrics, Dumfries Galloway Royal Infirmary, Dumfries, UK; 16 Clinical Research Facility, Royal Hospital for Sick Children, Glasgow, UK; 17 Department of Paediatrics, Raigmore Hospital, Inverness, UK; 18 Department of Paediatrics, Dr Grey's Hospital, Elgin, UK; 19 Department of Paediatrics, Perth Royal Infirmary, Perth UK

## Abstract

**Background:**

Gene-environment interactions are likely to explain some of the heterogeneity in childhood asthma. Here, we describe the methodology and experiences in establishing a database for childhood asthma designed to study gene-environment interactions (PAGES - **P**aediatric **A**sthma **G**ene **E**nvironment **S**tudy).

**Methods:**

Children with asthma and under the care of a respiratory paediatrician are being recruited from 15 hospitals between 2008 and 2011. An asthma questionnaire is completed and returned by post. At a routine clinic visit saliva is collected for DNA extraction. Detailed phenotyping in a proportion of children includes spirometry, bronchodilator response (BDR), skin prick reactivity, exhaled nitric oxide and salivary cotinine. Dietary and quality of life questionnaires are completed. Data are entered onto a purpose-built database.

**Results:**

To date 1045 children have been invited to participate and data collected in 501 (48%). The mean age (SD) of participants is 8.6 (3.9) years, 57% male. DNA has been collected in 436 children. Spirometry has been obtained in 172 children, mean % predicted (SD) FEV_1 _97% (15) and median (IQR) BDR is 5% (2, 9). There were differences in age, socioeconomic status, severity and %FEV_1 _between the different centres (p≤0.024). Reasons for non-participation included parents not having time to take part, children not attending clinics and, in a small proportion, refusal to take part.

**Conclusions:**

It is feasible to establish a national database to study gene-environment interactions within an asthmatic paediatric population; there are barriers to participation and some different characteristics in individuals recruited from different centres. Recruitment to our study continues and is anticipated to extend current understanding of asthma heterogeneity.

## Background

Asthma is a common condition diagnosed in as many as 25% of Scottish children by the age of 11 years[1]. Childhood asthma is heterogeneous in terms of severity[2], natural history [2] and response to treatment[3] and mechanisms for the disease heterogeneity among children with asthma is not well-understood but genetic and environment factors and, crucially, combinations thereof are thought to be relevant.

Asthma heterogeneity is most commonly classified by severity. Several genetic variations have been associated with asthma severity in children, including those within genes coding for filaggrin [4], macrophage inhibitory factor [5], interleukin-4 [6] and the beta 2 adrenoceptor [7]. Additionally, regulatory genes for ORMDL3 [8] and IL12B [9] have been associated with asthma severity. Environmental exposures have also been associated with asthma severity and include tobacco smoke[10] and outdoor air pollution[11]; there is also evidence of increased asthma severity in association with obesity[12] and reduced dietary antioxidants[13]. Whilst gene-environment interactions are described for asthma causation in children [14,15] there is also the potential for such interactions to explain why asthma severity varies within a population[16]. A proof-of-concept study[17] found that children with more severe asthma were more likely to carry at least one G allele for the CD14 gene but this is seen only among those exposed to tobacco smoke.

Large databases, for example BioBank UK http://www.ukbiobank.ac.uk and the Avon Longitudinal Study of Parents and Children (ALSPAC), have the potential to study gene-environment interactions in the context of asthma causation. However, understanding the relevance of gene-environment interactions to asthma heterogeneity will require a relatively large study population of children with asthma to provide enough statistical power to observe common interactions with a small effect and less frequent interactions which have a greater effect. Following a successful pilot of the methodology in Aberdeen, we have commenced recruiting patients under the care of consultant paediatricians with a special interest in asthma across Scotland. The aims of the Paediatric Asthma Gene Environment Study (PAGES) are:

1. To recruit children with asthma attending secondary care clinics

2. To ascertain the children's environmental exposures.

3. To obtain saliva samples consented for DNA preparation and genetic analysis.

4. To create a dataset sufficient to elucidate hypothesis-driven gene-environment interactions using a nested case-control design.

Initial gene-environment interactions of primary interest are between (i) exposure to tobacco smoke and dietary oxidants and genetic variations of the Glutathione S-transferase (GST) gene family and (ii) exposure to tobacco smoke and variations in the gene coding for filaggrin. With the advent of rapidly advancing technologies and the reduction in sequencing costs, we aim to combine the present population with the BREATHE cohort [4,18] and apply whole genome technologies to a population which is expected to number in excess of 2500 children and young adults with asthma.

The aims of the present report are to describe the methodology, demonstrate the feasibility of recruitment, describe our experience in establishing the database and report the characteristics of the children initially recruited.

## Methods

### Study design

**Figure 1:**
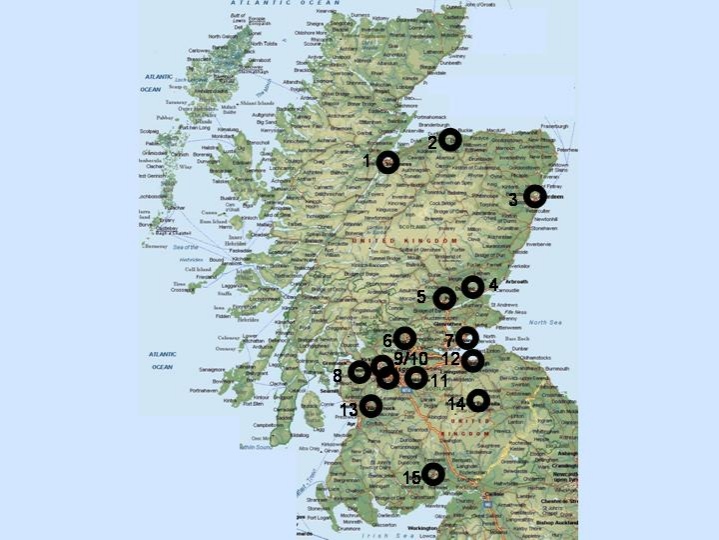
**A map of Scotland identifying the recruitment centres**. 1 = Inverness, 2 = Elgin, 3 = Aberdeen, 4 = Dundee, 5 = Perth, 6 = Stirling, 7 = Kirkcaldy, 8 = Paisley, 9/10 = Glasgow, 11 = Wishaw, 12 = Edinburgh, 13 = Kilmarnock, 14 = Melrose, 15 = Dumfries.

This is a cross-sectional study of asthmatic children attending hospital clinics across Scotland (figure 1) and Brighton, England (included as a validation centre). An asthma questionnaire is completed and returned by post. Children are invited to attend a clinical assessment that includes: spirometry, bronchodilator response, skin prick reactivity, exhaled nitric oxide measurement and saliva collection for cotinine assay and DNA extraction. Dietary and quality of life questionnaires are also completed during or after the clinical assessment A sample of saliva for DNA analysis can be collected by parents and returned by post in cases when clinical attendance is refused.

### Researchers

Researchers are based in five centres: Aberdeen (recruiting in Aberdeen, Inverness, Elgin, Perth and Dundee), Edinburgh (Edinburgh, Kirkcaldy, Melrose and Stirling), Glasgow (Glasgow, Wishaw and Paisley), Kilmarnock (Kilmarnock, Paisley and Dumfries) and Brighton. Researchers dispatch questionnaires, collect DNA and undertake the assessments in accordance with standard operating procedures and using identical apparatus.

### Eligibility criteria

Asthma is defined as a recurrent wheezing condition diagnosed as asthma by a consultant paediatrician who is a member of the Scottish Paediatric Respiratory Interest Group. All asthmatic children aged 2-16 years attending a clinic between March 2008 and November 2011 will be eligible. Exclusion criteria include children with coexisting respiratory morbidity, for example cystic fibrosis, bronchopulmonary dysplasia (BPD), and the following significant non-respiratory problems, cerebral palsy, Down's syndrome, gastro-oesophageal reflux (prescribed medications) and marked developmental delay. Children born prematurely but who did not have BPD are eligible; premature delivery is noted in the questionnaire.

### Enrolment

Our pilot study indicated that recruitment will not exceed 60% and therefore the gender, age and postcode of all children invited to participate are recorded to demonstrate how representative participants are of all the children invited to enroll. A deprivation index is derived from the postcode using 2009 Scottish Index of Multiple Deprivation data[19] (SIMD). Written consent is obtained from the parent when the asthma questionnaire is completed and verbal assent from the child at the time of the clinical assessment. This study has been approved by the Cornwall and Plymouth Research Ethics Committee

### Questionnaires

The asthma questionnaire (available at http://www.asthma-pages.com/participants/what/) included the respiratory questions validated in the BREATHE study[6], questions relating to asthma control (the Child Asthma Control Test^®^, used with permission) and environmental exposures (from Biobank). The Paediatric Asthma Quality of Life Questionnaire [7] and food frequency questionnaires (Scottish Collaborative Group semi-quantitative food frequency questionnaire version C1 http://www.foodfrequency.org) were also completed by parents.

### Clinical assessment

The assessment took place in conjunction with a scheduled clinic appointment or at a dedicated research clinic. The detailed assessment included (in this order) exhaled nitric oxide (FE_NO_), spirometry, skin prick reactivity and bronchodilator response. The assessment of children aged under 5 years included only skin prick reactivity since FE_NO _and reliable spirometry are often not obtained in this younger age group[20,21].

### Exhaled NO

Exhaled NO is measured using a portable NO analyser (NIOX MINO^®^, Aerocrine, Solna Sweden) in accordance with international recommendations[22]. Measurements from this device have been validated against a gold standard[20] and can be obtained in >90% in those aged over 7 years[20].

### Spirometry and bronchodilator response

These are measured in accordance with standard guidelines[23] using a portable spirometer (ML3500, MicroLab), calibrated before each assessment. Values are expressed as percentage of predicted according to normative data[24]. The bronchodilator response is defined as the change in FEV_1 _15 minutes after inhalation of 200 micrograms salbutamol, delivered from a pressurised metered dose inhaler via large volume spacer device (Volumatic^®^, GlaxoSmithKline, UK). Parents and children are asked to withhold short acting beta agonists for 6 hours prior to testing and long acting beta agonists for 12 hours.

### Skin prick reactivity

The standard methodology[25] is used to determine skin prick reactivity to eight common environmental allergens including: *Dermatophagoides pteronyssinus*, cat dander, dog dander, whole egg, *Alternaria alternans*, *Aspergillus fumigatus*, peanut and grass (ALK, Northampton). The positive control is histamine 10 mg/ml and the negative control 0.9% saline. A positive skin test is defined as a weal ≥3 mm in longest diameter or, in cases of dermatographism, greater than the negative control. Testing is withheld in individuals who have taken anti histamines within the previous 72 hours. Testing for peanut is withheld in children with a history of peanut anaphylaxis. Children with a positive reaction to peanut but no history of reaction after ingestion of peanuts are offered referral for peanut challenge.

### DNA collection, storage and analysis

Oragene sampling kits (DNA Genotek Inc, Ottowa, Canada), were used to obtain DNA from a saliva specimen. Five swabs (DNA Genotek Inc, Ottowa, Canada) are used to collect saliva for DNA analysis in young children who cannot actively provide a saliva sample. Saliva is either obtained by the researcher during a hospital visit or occasionally by the parent at home and then mailed to the researcher. Saliva samples are immediately labeled using preprinted barcode labels which are also attached to the case report form. This provides an immediate and robust de-identification system to retain the privacy of the study subjects, but retaining the ability to link genotype to phenotype within the study.

### Salivary Cotinine

Saliva is collected for cotinine assay using a sterile absorbent cotton wool swab (Salivette^®^, Sarstedt Ltd, Leicester, UK) which is placed between teeth and buccal mucosal for three minutes before being inserted into a plastic case. Specimens are spun down and saliva frozen for analysis by ELISA (ABS laboratories, Welywn Garden City, UK). Cotinine exposure is analysed as a continuous variable and values of ≥15 ng/ml assumed to indicate active smoking by the child. Values below the limit of detection (0.1 ng/ml) are assigned a value of 0.05 ng/ml.

### Establishment and population of the database

**Figure 2:**
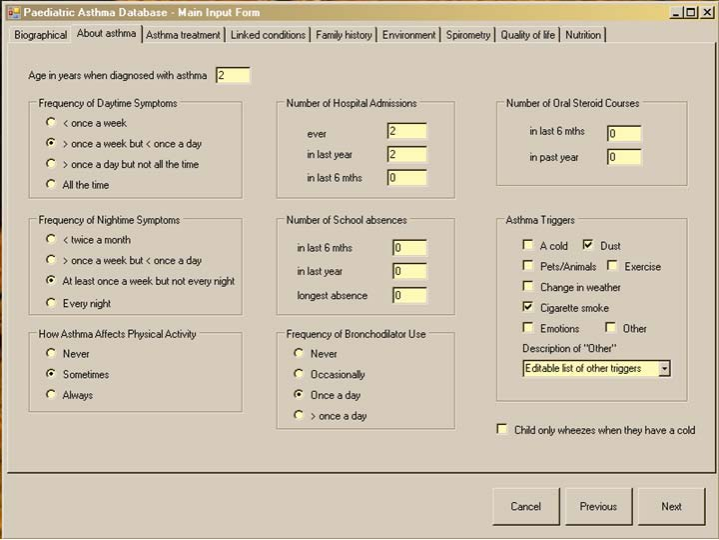
**A view of one of the screens on the database**.

The experience gained from establishing and validating the UK CF database[26] has been applied to the PAGES database. The database is currently a stand alone (non-networked) system (figure 2). There is the option for future remote, web based access to the database now that the fields are fully agreed and the methodology is stable. The advantage of using a database system for development (rather than working with a spreadsheet or statistics package) is that database systems naturally facilitate the inclusion and preparation for the analysis of longitudinal data. Standard validation checks have been incorporated as described in detail elsewhere[26,27] and include restricted fields, e.g. age is a numeric field between 2 and 16, gender is a categorical field either "male" or "female".

### Quality control

To ensure a standardised methodology all researchers work to the same standard operating procedure and use identical equipment. Researchers have a certificate from Association for Respiratory Technology and Physiology/British Thoracic Society; this is a national qualification for those practitioners who complete the Spirometry assessment. All researchers attend a monthly minuted teleconference where methodological issues that arise are discussed and resolved. Finally, all data are entered onto one central database by an individual. All queries or errors identified at the verification stage are notified back to the researcher within 2 weeks of receipt; this approach is proven to result in data entry of high quality[26]. An audit of data is undertaken in the course of the present analysis.

### Power calculation

Power calculations are based on the pilot study. The sample size needed to detect the interaction of a genetic factor with an exposure depends on the prevalence of exposure and genotype, the relative risk for exposure and genotype alone, magnitude of the gene-environment interaction, the case-control ratio and the type I and type II error [28]. For example to explore the relationship between tobacco smoke exposure (assuming 50% prevalence) and GSTT null genotype (assuming 50% prevalence) and the presence of atopic illness as an outcome among an asthmatic population, assuming relative risks of interaction (Ri) of 2 and 5, studies including 414 and 103 non-atopic asthmatics respectively will have power of 80% to detect an interaction at 5% level of significance [29]. To relate an interaction between filaggrin variants (assuming 10% prevalence) and second hand smoke exposure (assuming 50% prevalence) with asthma severity, assuming relative risks of interaction of 2 and 5, studies including 199 and 105 asthmatics on British Thoracic Society (BTS) treatment steps 4 and 5 respectively will have power of 80% at 5% level of significance[29].

### Statistical approach to analyzing the final dataset

Statistical analysis will be conducted using logistic regression which permits adjustment for confounders, and both crude and adjusted odds ratios will be presented. To test for gene-environmental interaction, an interaction variable will be created based on the combination of factors investigated[30]. First the model will be fitted with both variables as separate term, then with an interaction term. The statistical significance of the interaction will be obtained from likelihood ratio test of change in deviance between the second model and the first.

### Statistical approach to present report

Differences between centres were compared using chi square, Mann Whitney-U test and Kruskal Wallis test as appropriate. Significance is assumed at p = 0.05. Standard statistical software is used (SPSS version 17.0.0)

## Results

### Recruitment

**Figure 3:**
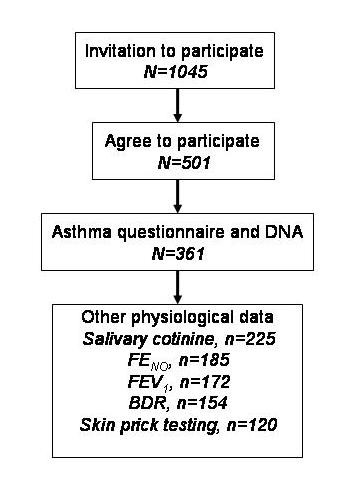
**A Consort diagram demonstrating the number of individuals where data are available**. Recruitment is ongoing and these numbers will increase before the end of the study.

At the time of writing 1045 children have been invited to participate (figure 3), of whom a DNA sample has been obtained in 436 (42%) and asthma questionnaire in 382 (37%); the discrepancy in numbers where DNA and questionnaire were obtained is in part explained by many of the latter currently being entered onto the database. Recruitment has been underway for more than 12 months in seven centres (listed in table 1), for more than six months in nine centres and for more than one month in fourteen centres. An audit of the database found several discrepancies between treatment and BTS/SIGN treatment step; the error is not at the level of data entry rather at the level of the researcher coding the treatment step. The audit found no outliers and other than BTS/SIGN step, data were ready for analysis.

**Table 1:** Comparison of participation rates, ages and asthma outcomes between centres.

Centre	Number of children		Mean age (years)		Median SIMD decile		**Mean % FEV**_**1**_	**Median FE**_**NO **_**(SEM), ppb**	Median BTS treatment step
				
	Invited	Participated	Participants	Non participants	Participants	Non participants			
Aberdeen	167	93	8.8 (4.1)	9.1 (4.3)	7	7	96 (17)	27 (6)	3
							n = 41	n = 41	

Dundee	220	91	8.1 (4.2)	9.0 (4.4)	6	5	100 (17)	25 (6)	3
							n = 45	n = 44	

Edinburgh	271	140	8.5 (4.1)	9.8 (4.3)	7	5	91 (11)	16 (4)	3
							n = 38	n = 54	

Elgin	70	29	8.9 (4.1)	9.8 (4.3)	6.5	5	103 (12)	17 (6)	3
							n = 12	n = 9	

Kirkcaldy	80	42	6.8 (3.3)	8.5 (3.6)	5	4	93 (14)	11 (18)	3
							n = 9	n = 8	

Melrose	24	17	11.1 (3.0)	10.5 (3.8)	7	5	93 (9)	14 (7)	3
							n = 13	n = 15	

Perth	119	45	10.0 (3.4)	9.8 (3.7)	6	7	104 (12)	41 (10)	3
							n = 14	n = 14	

Other	94	64	9.0 (4.3)	8.3 (4.3)	4	4	none	None	2.5

Overall	1045	501	8.6 (3.9)	9.2 (4.0)*	6 †	6	97 (15)†	18 (2)	3 (2, 3)†

SIMD = Scottish Index of Multiple Deprivation, SEM = Standard Error of Mean, BTS = British Thoracic Society.*p = 0.022 for comparison between participants and non participants. † p value for trend tests for %FEV_1_, SIMD decile and BTS severity between centres <0.05.

### Reasons for non-participation

These were prospectively ascertained as part of the pilot study where 91 children were invited to participate of whom 57 (62%) were enrolled. Of the 34 children who were not enrolled, the parents of 17 did not have time to participate during the clinic visit, 10 did not attend the scheduled clinic appointment, five refused to take part and two children did not have asthma. When the centres recruiting for less than a year were combined into an eighth group, inter centre recruitment rates varied between 38% and 72%, p = 0.004, table 1.

### Factors associated with participation

For the purpose of the present study, participation is defined as at least a DNA sample or asthma questionnaire being obtained (n = 501). The mean age (standard deviation) of those enrolled is 8.6 (3.9) years and 9.2 (4.0) years for those who did not enroll (T test p = 0.022). There were 288 boys recruited (57% of all enrolled) and 359 boys (64%) among those who did not enroll (χ^2^_1 _= 4.71, p = 0.030). The median (range) SIMD decile was the same (6 (1, 10)) for those who did and did not enroll (Mann Whitney U test p = 0.135).

### Differences between centres

These details are presented in table 1. The centres where recruitment has been under way for less than a year were combined to form an eighth group. There were significant inter-centre differences in the age of children invited to take part (p = 0.002), SIMD (p < 0.001), BTS treatment step (p = 0.002), % predicted FEV_1 _(p = 0.024) but not gender or FE_NO_.

### Participant details

The mean age (SD) was 8.6 years (3.9) and 57% were boys. There were 118 children aged under five years including 24 aged between two and three years and 55 aged three to four years. The proportions of children on each British Thoracic Society treatment step were: 29% step 2, 60% step 3, 10% step 4 and 1% step 5. Saliva was collected for DNA analysis in 436 and for cotinine assay in 225. Spirometry was measured in 172 children, mean (SD) % predicted FEV_1_, FVC and FEF_25-75 _were 97% (15), 106% (13) and 79% (27) respectively. Bronchodilator response was measured in 154 children and the median (interquartile range) was 5% (2, 9). Exhaled NO was measured in 185, median (IQR) value 18 (12, 50) parts per billion. Skin prick reactivity was measured in 120 children including 99 who were reactive. Reasons for missing pulmonary function data include young age, refusal to provide measurements and inability to provide measurements of adequate quality. Reasons for missing skin prick reactivity data include recent receipt of antihistamines, previous peanut anaphylaxis and refusal to have testing. There were no significant associations between BTS treatment step (severity) and physiological outcomes including spirometry, BDR or FE_NO_.

### Salivary cotinine validation of tobacco smoke exposure

Salivary cotinine concentration was determined in 139 children and was in excess of 15 ng/ml in six individuals (median age 14.5 years). The median (SEM) cotinine for children with no resident smokers was 0.5 ng/ml (0.04), with one resident smoker was 0.58 ng/ml (0.16) and with two resident smokers was 1.39 (0.95), Kruskal Wallace test p < 0.001.

### Different administrative requirements between centres

Although the study is categorised site-specific exempt by the ethics committee, three centres still requested Site Specific Assessment documents. One centre requested a materials transfer agreement with the host university. Some centres, but not all, required visiting researchers to have honorary contracts. In some centres, approval documents are sent initially to Research and Development departments whereas in others these documents go directly to the local Research Ethics Committee.

## Discussion

Here we describe the methodology and feasibility for recruiting children with asthma on a national basis. To our knowledge this is the first study specifically designed to study asthma heterogeneity in children rather than asthma causation which has been the focus of much research interest. In addition to describing our methodology, we provide novel findings including barriers to participation in asthma research, details of passive smoke exposure in children with asthma and differences in the characteristics of children with asthma recruited from centres across the nation. These experiences may be relevant to colleagues interested establishing national and international single-disease databases in other countries.

The potential for national single-disease databases to study respiratory disease has already been demonstrated in cystic fibrosis. Cystic fibrosis (CF) databases have been established in several countries including the UK [31] and have lead to a better understanding of disease heterogeneity [32,33] and also added to our understanding of genetic mechanisms[34]. Asthma is more prevalent than CF and an asthma diagnosis is made on a solely clinical basis but notwithstanding these important differences between asthma and CF, we believe that the known benefits of national CF databases will be applicable to the present asthma database.

We are not aware of pre-existing large single-disease asthma databases from which the relationship between asthma heterogeneity and gene-environment interactions can be studied, but cross-sectional and longitudinal studies of asthmatic populations in Europe [18,35], United States[36] and Australia[2] have provided important insights into the heterogeneity of the natural history of asthma. A number of publications from the Childhood Asthma Management Programme[36] (a randomised-controlled trial including 1041 asthmatic children with follow up for over four years) have demonstrated associations between genetic variations and heterogeneity in response to asthma treatment [37-39]. A study of the 5244 asthmatic children who took part in the whole-population NHANES III survey in the US also found evidence for asthma heterogeneity in terms of both risk factors and severity [40]. These studies[2,18,35-40] give some insight into the heterogeneity of childhood asthma and the present study seeks to explore the variability described.

One challenge to our study is the absence of gold standard for measuring asthma heterogeneity outcomes. Severity is most commonly outcome used and in adult populations, indices of asthma severity have been developed and validated for use in large databases[41]. Based on our prior experiences [4,18] we will use BTS treatment step as our primary heterogeneity outcome. Once the dataset is complete we will explore the utility of other severity outcomes, for example asthma control, FEV_1_, FE_NO_, hospitalisations and the number of courses of oral steroids over the past year. Additionally, we will explore heterogeneities in treatment response, i.e. bronchodilator response, and symptom triggers in the context of plausible gene-environment interactions.

Our methodology has a number of strengths, weaknesses and limitations. A key strength is that we have demonstrated that our methodology captures a heterogeneous population in terms of severity, as evidenced by BTS treatment, skin prick reactivity, exhaled nitric oxide and spirometry. A second strength is that phenotyping is carried out by researchers who use standardised methodology in all centres; this approach to quality control minimises the risk of heterogeneity arising between centres due to methodological differences. A third strength is that we have validated second hand smoke exposure, one of our principle exposures of interest, with a biomarker. A weakness of the study is the low recruitment rate, which at present is less than 50% and, as evidenced by our pilot study, is not expected to exceed 60%. We have described the barriers to participation and these are not previously described in the context of asthma and include pressure on parent's time but uncommonly outright refusal to participate. An intervention study of behaviour problems in children has also reported time demands explaining 50% of non-participation[42]. Despite this relatively low recruitment rate, our study is likely to be adequately powered; for example we have already recruited more than half of the 105 children on BTS treatment steps 4 and 5 required to explore interactions between filaggrin gene variants and ETS. A second weakness is that spirometry, exhaled NO and skin prick reactivity data have not been obtained in individuals where exposure and genetic date are available; reasons for non-collection of clinical data include young children not being able to provide spirometry and exhaled NO data and skin reactivity data missing in those with peanut anaphylaxis. One limitation to this study is its cross sectional nature and we will seek funding to follow up those recruited to study disease remission and persistence over time. We acknowledge that the present study is hospital-based and the results may not be relevant to all children with asthma in the community. A further potential limitation to the study is inclusion of children as young as two years since asthma and viral induced wheeze are prevalent in preschool children and distinguishing between the two conditions can be difficult. The proportion of preschool children with recurrent wheeze is greater than in older children [43] but in the present study, preschool children were in the minority which might suggests that our inclusion criteria may be excluding children with non-asthmatic preschool wheeze; in our final analysis we will consider the whole dataset and then exclude the preschool group to address this limitation.

Assessment of environmental exposures is not without difficulties in such studies as this. Our methodology includes salivary cotinine as an objective index of exposure to second hand tobacco smoke (SHS) and we have observed higher exposure among those with greater reported exposure. The concentrations of salivary cotinine are lower than those in an earlier study undertaken in Dundee [44] where the average concentration in asthmatic children where two adults smoked is 4.02 ng/ml and 1.62 ng/ml where only mother smoked. This apparent fall in salivary cotinine concentrations may represent a genuine reduction in exposure of asthmatic children to SHS between 1994 and the present time or may be due to parents in the present study reducing their child's exposure to SHS prior to the assessment knowing that cotinine is to be measured. Questionnaire information on factors such as smoking, parental smoking, use of gas fires and gas hobs and open fires is usually reasonably robust and memory of past exposures by the parents will be reasonable as the time scale is relatively short in comparison with studies of historical exposures in adults. The child's home address will provide the ability to model outdoor air pollution exposure and future secondary studies could directly assess of indoor air quality, for example concentrations of particulate matter (PM_10 _and PM_2.5_), relevant gases (notably oxides of nitrogen) and volatile organic compounds. Exposure to other factors such as chemicals in the home known to be associated with respiratory symptoms[45] is less clear cut but the development of this database would allow additional studies to be added to the core concept (much as has been done in ALSPAC[45]) to allow specific issues to be addressed with more specific measurement of any relevant environmental exposures.

In summary, our study aims to provide a dataset from which to explore the influence of gene-environment interactions in childhood asthma. The gene-environment interactions associated with asthma causation will not necessarily explain asthma heterogeneity and we hope to report novel insights into genetic variations within an asthmatic population. Although the primary purpose of the study is to further understanding of asthma heterogeneity, there are other potential applications of the present study and these are listed in table 2.

**Table 2:** Potential applications of a national asthma database.

Potential application	Resource
Identification of genetically susceptible individuals for pharmacogenetic studies	DNA

Identification of genetically susceptible individuals for environmental modification studies	DNA

Characterisation of patterns of inhaled environmental hazards in children with asthma	Questionnaire and longitudinal component

Validate a system to score asthma severity	All data

Explore gene-environment interactions for subgroups, eg severe asthma, non-atopic asthma	Subgroups

Audit standard of care between centres	Management + CACQ

Identify associations between gene-environment interactions and natural history	Longitudinal study

Confirm associations seen in secondary care study	Primary care study

## Conclusions

We conclude that it is feasible to study gene environment interactions in children with asthma by recruiting from multiple centres. There are differences in participation rates and patient characteristics between centres. The main reasons for non-participation in such a study were lack of time on parent's behalf and failure to attend clinic appointments.

## Competing interests

The authors declare that they have no competing interests.

## Authors' contributions

SWT, JGA, TVM, AM, SM and CNAP conceived of the study, and participated in its design and coordination. SWT, SM, SC, TA, KA, DC, AD, GH, UM, JMcC, AM, AW and SW administered the study at each site thereby allowing data acquisition. CB, RL, DM and EW undertook recruitment and assessment. SMcL undertook the pilot study. All authors read and approved the final manuscript.

## Pre-publication history

The pre-publication history for this paper can be accessed here:

http://www.biomedcentral.com/1471-2288/10/107/prepub
